# 

**Published:** 1894-07-14

**Authors:** 


WITH EXTRA NURSING SUPPLEMENT.
Per Annum, 10s. 6d., post free.
Monthly Parts, 6d.; post free, 8d.
Ditto per Annum, 8s. 6d., post free.

### THE HOSPITAL

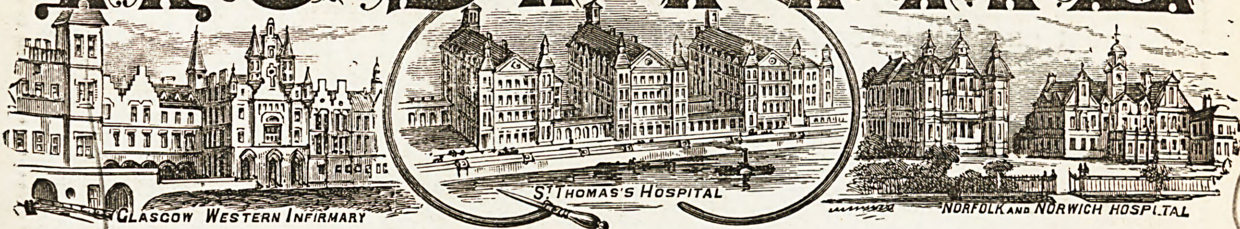

No. 407. Vol. XVI. WITH EXTRA NURSING SUPPLEMENT. SATURDAY, JULY 14TH, 1894.

